# Preventing Fungal Spoilage from Raw Materials to Final Product: Innovative Preservation Techniques for Fruit Fillings

**DOI:** 10.3390/foods13172669

**Published:** 2024-08-24

**Authors:** Teresa Bento de Carvalho, Beatriz Nunes Silva, Elisabetta Tomé, Paula Teixeira

**Affiliations:** Universidade Católica Portuguesa, CBQF—Centro de Biotecnologia e Química Fina—Laboratório Associado, Escola Superior de Biotecnologia, Rua Diogo Botelho 1327, 4169-005 Porto, Portugal; s-mtmcarvalho@ucp.pt (T.B.d.C.); bnsilva@ucp.pt (B.N.S.); etome@ucp.pt (E.T.)

**Keywords:** mycotoxins, food waste prevention, fungi, yeast, molds

## Abstract

Spoilage fungi are a significant cause of financial loss in the food and beverage industry each year. These fungi thrive in challenging environments characterized by low acidity, low water activity and high sugar content, all of which are common in fruit fillings used in pastry products. Fruit fillings are therefore highly susceptible to fungal spoilage. Fungal growth can cause sensory defects in foods, such as changes in appearance, odor, flavor or texture, and can pose health risks due to the production of mycotoxins by certain mold species. To reduce food loss and waste and extend product shelf-life, it is critical that we prevent fungal spoilage. Synthetic chemicals such as sorbic acid and potassium sorbate are commonly used as preservatives to prevent fungal spoilage. However, with consumer demand for ‘natural’ and ‘chemical-free’ foods, research into clean-label preservative alternatives to replace chemical preservatives has increased. The objectives of this review are (i) to provide an overview of the sources of fungal contamination in fruit filling production systems, from pre-harvest of raw materials to storage of the final product, and to identify key control factors; and (ii) to discuss preservation techniques (both conventional and novel) that can prevent fungal growth and extend the shelf-life of fruit fillings.

## 1. Introduction

Food spoilage is a major concern in the food industry, resulting in significant economic costs due to loss of product before reaching the retail stage or due to food recalls, the latter having a tremendous impact on consumer opinion and trust in food companies and retailers. Moreover, subsequent food waste poses considerable issues, not only from social and humanitarian perspectives but also from an environmental standpoint [1].

Filamentous fungi are among the most common spoilage microorganisms in the food industry, found throughout the production process, from raw materials to finished products, even on highly processed and stable products [2]. Fungal spoilage is a common and widespread problem, particularly in pastry manufacturing, considering that fruit fillings, purées and jams are widely used, and their high sugar content and water activity are promoters of fungal growth. Fungal growth and, consequently, the production of exoenzymes can result in impaired organoleptic properties such as unpleasant odor, texture, and flavor. Additionally, visual changes may occur, with noticeable colony growth on the contaminated product [1,3].

In shelf-stable hot filling products such as fruit fillings, the most commonly found and identified spoilage microorganisms belong to the species *Penicillium*, *Aspergillus*, *Cladosporium*, and *Zygosaccharomyces* [1,4]. *Penicillium* and *Aspergillus* are known for their ability to produce secondary metabolites, namely, mycotoxins, which are toxic compounds produced by molds that are hazardous to humans [5]. The main threat is that although the mycotoxigenic spoilers may no longer be detected, the mycotoxins may still be present, as they are often resistant to the employed control strategies, including heat treatments [6]. In other cases, molds may secrete mycotoxins into the foodstuff without visible signs of spoilage that would alert the consumer to reject the food product [7]. The most important mycotoxins include aflatoxin, ochratoxin A and patulin, but others such as fumonisins and trichothecenes may also be present, depending on the fungal contamination of the food [8,9]. For example, aflatoxins are derivatives produced by many strains of *Aspergillus flavus* and *Aspergillus parasiticus*; ochratoxin A is mainly produced by *Aspergillus* and *Penicillum* species [10]; and patulin is also produced by numerous species of *Aspergillus* and *Penicillum*, but also by *Byssochlamys nivea* [11]. In turn, fumonisins are produced by various *Fusarium* species [8], and trichothecenes are produced by *Trichoderma*, *Fusarium*, *Stachybotris*, and *Myrothecium* genera [12].

The occurrence of fungal spoilage at any stage of the food supply chain is a major concern, as yeasts and molds may be able to overcome control strategies already in place, including low pH and water activity, added preservatives, oxygen limitation, and thermal processing [4]. Nonetheless, spoilage fungi are typically introduced to industry environments through contaminated raw materials. Hence, tracking the entire manufacturing process with multiple checkpoints, from raw material acquisition to composition, formulation, processing, packaging, and storage, is crucial for ensuring the quality and safety of the finished product. This approach also helps to reduce the probability of spoilage growth and propagation between environments [4]. More specifically, stringent ingredient quality specifications, optimal processing conditions, safe handling and well-established quality control measures, implementation of Hazard Analysis Critical Control Point (HACCP) system, and comprehensive sanitation programs are essential to control food spoilage microorganisms [4].

Mycotoxin-producing fungi and other spoilage microorganisms can also be deterred by chemical fungal inhibitors, which include calcium and sodium propionate, sorbic acid, potassium sorbate, sodium diacetate, sodium benzoate, and acetic acid [7,13]. Albeit chemical additives such as potassium sorbate, propionates and benzoates are the most effective antimicrobials against fungi in bakery products with fillings, consumer demand for clean-label replacements has prompted the need to investigate natural alternatives [14].

**Figure 1:**
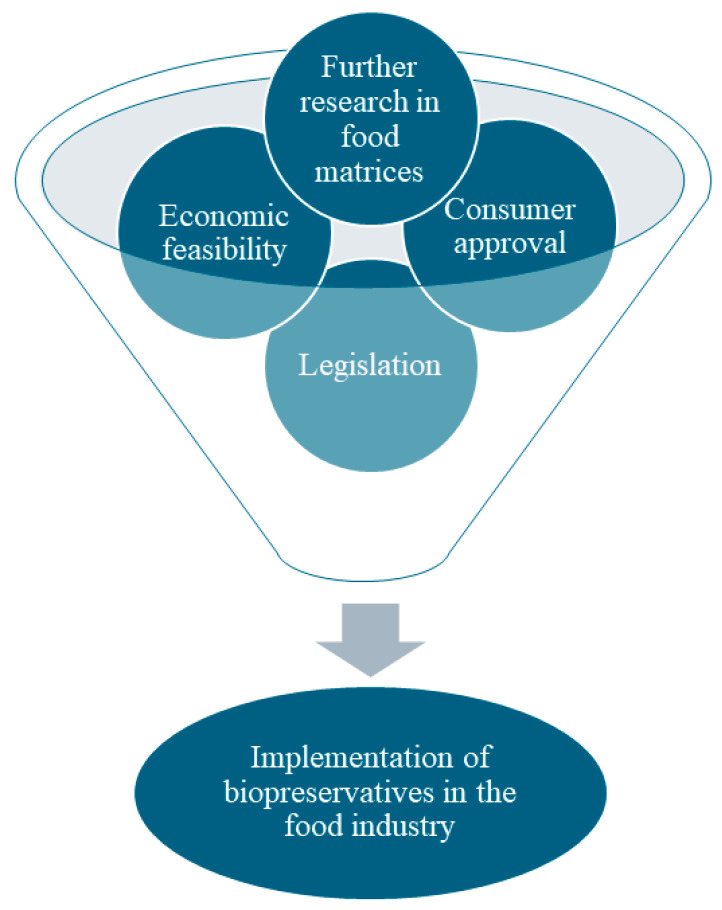
Factors affecting the implementation of biopreservation approaches by the food industry.

There is no official definition of “clean label”, as it is not a scientific term, but it generally means a label with a short and simple list of ingredients that consumers can understand and perceive as natural and organic, excluding substances that have a negative connotation due to their unfamiliar names, such as e-additives [15]. Clean label alternatives to synthetic chemical preservatives may be derived from animal, plant, or microbial sources. From chitosan to spices, essential oils, fruit and vegetable extracts, vanillin, bacteriocins and fermentates, many clean-label alternatives have proven to be an efficient control measure in food products [1,14,16,17]. Nevertheless, the widespread implementation of these biopreservation approaches still depends on factors concerning economic feasibility, consumer approval, additional research in food matrices, and legislation that can act as significant obstacles to their adoption (Figure 1).

Considering the impact of fungal spoilage on pastry fruit products, this review aims to (i) provide an overview of the sources of fungal spoilage in fruit filling production systems, from pre-harvest of raw materials to storage of the final product, identifying crucial control factors, and (ii) present and discuss the currently available conventional and clean-label preservation strategies for preventing fungal growth and extending the shelf-life of fruit fillings. The main problems associated with fungal spoilage of fruit fillings, as well as the traditional and clean label control measures currently available will be highlighted, with a focus on the use of natural antimicrobial compounds for shelf-life extension and preventing fungal spoilage.

## 2. Main Fungal Spoilage Issues Associated with Pastry Fruit Fillings

The susceptibility of pastry fruit products to fungal spoilage depends on many factors [1,18], including (i) the characteristics, composition, and biological parameters of the food matrix (such as rheology, texture, nutritional composition and indigenous microbiota) and the food’s physicochemical parameters (e.g., pH, water activity, titratable acidity, total soluble solids, i.e., Brix); (ii) raw material collection, handling and storage before, during, and after harvest (temperature, hygrometry, safe handling of produce); (iii) the product manufacturing process; and (iv) storage of the final product (packaging conditions such as temperature, relative humidity and atmosphere). The combination of food matrices and extrinsic factors favorable to fungal growth (e.g., temperature and humidity) facilitates the proliferation of fungal spoilage in foods [1]. This implies that such food matrices may need to be modified to hinder spoilage growth, or, alternatively, antifungal measures must be taken. The following sections delve into the factors enumerated above.

### 2.1. Matrix Characteristics and Physicochemical Parameters of Fruit Fillings

Fruit fillings are mainly composed of fruit-based raw materials, water, sugar, and hydrocolloids, which serve as stabilizing, emulsifying, thickening and gelling agents [18,19], since gelation is essential for regulating texture in fruit fillings [20]. Xanthan gum, gum Arabic, guar gum, carboxy methyl cellulose, locust bean gum, κ-carrageenan, alginate, and pectin are among the several hydrocolloids with gelling properties that are available to produce fruit fillings with improved stability and texture for the pastry industry [21,22]. Moreover, hydrocolloids can help to reduce the use of sugar in fruit fillings while maintaining the desired sensory properties [21], which results in products with improved nutritional profile and less sugar available as a carbon source for fungal spoilage. Reduced-sugar fruit fillings may also be obtained using clean-label ingredients other than hydrocolloids. Carcelli et al. [23], for example, successfully produced a 30% sugar-reduced fruit filling, which was undistinguishable from the control in terms of sensorial attributes, using a fiber syrup based on corn dextrin and the seed coating of chickpeas. On the other hand, Agudelo et al. [24] compared fruit fillings made with sucrose to fruit fillings reformulated with polydextrose and intense sweeteners and found that the replacement of sugar led to product rejection due to the intense taste of the sweeteners. This illustrates the complexity of product reformulation, which is designed to meet consumer demand but can result in the creation of a product with unappealing sensory attributes that consumers will reject.

To be used in bakery fillings, hydrocolloids must display adequate pumpability prior to baking and provide stability to the final product, which is achieved when they can preserve their shape after baking; they must also display low syneresis, i.e., reduced expulsion of liquid (water) from the gel [20]. Alginates and pectin, for example, have been shown to provide bake-stable functioning in a variety of applications, including fruit fillings [20].

With regard to stability, fruit fillings intended for use in bakery products must be designed to be stable at both high and low temperatures, as they must endure heating during the filling preparation and baking processes as well as chilling and/or freezing during cold storage [19]. In this sense, although it may be challenging to keep frozen or refrigerated products at a constant, ideal low temperature, repeated freeze–thaw cycles throughout the supply chain must be avoided, as they may result in syneresis and altered, undesirable, rheological properties [25]. In fact, when baked or kept frozen, fillings that are not thermally stable tend to deteriorate inside the pastry dough, causing water to be released from the gel formed to the outside of the dough, making the product unappetizing and compromising its integrity [19].

Fungi, like all other microorganisms, are strongly affected by water availability and pH. For pastry products, it is essential to target specific pH and water activity values for fruit fillings, as the addition of fruit pulp or pieces to pastry products influences the rheological characteristics and texture of the finished product, as described by Agudelo et al. [25]. In particular, the pH of food filling systems plays a critical role in influencing the ion-binding properties of many hydrocolloids, thereby increasing or decreasing their gelling behaviour [21]. In this sense, the degree of impact varies depending on several factors, including the pH of the fruit pulp or pieces, the amount of fruit added, the type of fruit, the size of the fruit purée particles, the soluble solids content, the concentration of hydrocolloids, and the interactions between these components [25].

The final moisture content and water activity of fruit fillings are linked to the technological processes used for their production [1]. Fruit fillings generally present high moisture content, relatively high water activity, and high acidity [22], which together with the content in soluble solids (Brix) have a major impact on the color, flavor, aroma, texture, and microbial stability of the final product [18]. For example, Wei et al. [22] reported that the pH values of apple, blueberry, raspberry, and lemon-based fillings ranged from 2.6 to 3.5, while the soluble solids content was reported to be higher than 34 °Bx, with fruit fillings usually containing less than 55% of sugar [26].

### 2.2. Raw Materials (Pre- and Post-Harvest)

Fresh produce, such as fruit, is highly perishable and spoils quickly after harvest for a variety of reasons, most of which are pre-harvest related. Fruit quality and shelf-life are highly impacted by climatic conditions, relative humidity, cultivation practices, type of soil, irrigation water, fertilizer used, amongst others. Fruit type and condition (for example, its maturity), also play an important role in pre-harvest fungal diseases or predisposition to post-harvest decay [27,28,29].

As reported by Matrose et al. [30], fungal infection can occur during pre- or post-harvest. During pre-harvest, it can occur on the surface of floral parts or on the developing fruit, and during post-harvest, it can occur due to lesions to the fruit surface, caused by insects, operators’ handling, or machine harvesting [27,28,30]. In fact, fruits with physical injuries such as bruises, abrasions, wounds, senescence, or defects caused by pests are more susceptible, considering that fungal spores can find the right conditions for their development within these injuries [28].

As the post-harvest period involves several stages, i.e., harvesting, handling, selection, washing, sorting, and packing followed by transport and storage, the post-harvest system is likely to affect the shelf-life of the fruit as well as its appearance, taking into account damage due to mechanical causes, which are most often bruises, dents, and cuts/scars on the products [27,28]. In fruits, most post-harvest losses are due to fungal spoilage, mostly caused by filamentous fungi, and the visual signs of contamination vary depending on the saprophytic molds growing on the surface of the fruit [28].

Matrose et al. [30] have identified the main fungal genera associated with relevant food spoilage as *Aspergillus*, *Botrytis*, *Penicillium*, *Rhizopus*, *Mucor* and *Alternaria* (Table 1). In turn, *Pichia*, *Zygosaccharomyces*, *Saccharomyces*, *Candida* and *Rhodotorula* are reported as the main yeast genera associated with spoilage of processed fruit products (Table 2).

Post-harvest spoilage of fresh fruit, including apples, pears, strawberries, mangoes, and peaches, is most often caused by *Aspergillus niger*, which is the most prevalent *Aspergillus* species in post-harvest diseases [18]. Furthermore, *Aspergillus* species are known to produce mycotoxins, namely aflatoxin and ochratoxin A, and, to a lesser extent, patulin [41]. Although mycotoxin production has not been associated with *Botrytis* [18], this genus also causes decay in a wide variety of fresh fruits. It is the leading cause of spoilage in berries, including strawberries, blueberries, raspberries, and blackberries, and the major cause of disease in grapes, both pre-harvest and post-harvest. It is also the cause of significant losses in apples, pears, and tomatoes [18]. *Penicillium* species, particularly *Penicillium expansum*, are often responsible for blue mold in apples, a serious post-harvest disease accountable for most apple losses worldwide [42]. Moreover, this fungus can produce harmful mycotoxins such as patulin and citrinin [43]. *Rhizopus* spores are ubiquitous in agricultural environments and, due to their high incidence, often cause soft rot as a post-harvest disease [44,45]. *Rhizopus* rot has been found on strawberries, peaches, tomatoes, pumpkins, and dates [6,44,46]. In addition, the presence of endofungal bacteria (“bacterial symbionts of fungi that exist within fungal hyphae and spores”, as defined by Alabid et al. [47] in some strains of *Rhizopus* species, which can produce mycotoxins such as rhizonin A and rhizonin B, is another reason to monitor this genus and its mycotoxin production potential [48].

Similar to *Rhizopus*, *Mucor* spp. also causes soft rot during post-harvest, and both genera mainly infect damaged fruit [49]. *Mucor* species are of potential concern, with some species having mycotoxin production potential [33].

Finally, the genus *Alternaria* includes fast-growing spoilage fungi found mainly during post-harvest processes and storage (but also before harvest [50,51]) and has been detected in apples, tomatoes, blueberries and cherries [50,51,52,53]. *Alternaria* spp. produce several mycotoxins, of which tenuazonic acid is the most relevant due to its harmful effect on eukaryotic protein synthesis and mutagenic potential [18,54].

Spoilage fungi can enter the manufacturing chain of fruit fillings by means of contaminated fruits that were not identified and discarded at any quality checkpoint. Even though it is likely that the mycelium will be eliminated by processing and heating of the fruit purée, spores and mycotoxins that may be present will remain in the finished product. These are major issues, considering that spores may germinate later in the final product, spoiling it and leading to visible spoilage; on the other hand, the presence of mycotoxins is problematic because they may not be detected on the product through visual inspection or other sensorial means, meaning that the product may be consumed and cause severe illnesses. Aflatoxins are known for their genotoxicity; ochratoxin A produces nephrotoxic, hepatotoxic, immunotoxic, genotoxic, teratogenic and neurotoxic effects, and fumonisins have teratogenic and immunotoxic effects and have been associated with neural tube defects and oesophageal cancer in humans [9]. Patulin, in turn, causes mainly gastrointestinal disorders—namely, bleeding, distention, and ulceration—and leads to congestion and oedema (fluid accumulation causing swelling of the affected tissue) of the gastrointestinal, hepatic, and pulmonary blood vessels and tissues [9].

Naturally occurring yeast populations in raw materials are predictably one of the most common sources of contamination in processed foods and beverages. Knowledge of the microbial community in fresh fruit, where epiphytic yeasts predominate, is particularly important in fruit-based products [38]. Food spoilage yeasts, which develop under extreme conditions, spoil fresh and canned fruits and products derived from them, such as juices and purées, soft drinks, and wine [37,38]. Signs of product spoilage include softening, development of off-flavors and off-odors, and undesirable ethanol production due to the presence of fermentative yeasts [55]. Yeast development thrives in environments with high sugar levels of 40–70%. Osmotolerant yeasts such as *Zygosaccharomyces bailii* and *Zygosaccharomyces rouxii* are the main causes of spoilage in fruit products, fruit juices, syrups, and honey, growing visibly on the surface of packaged products. Bevilacqua et al. [37] reported that *Saccharomyces* spp. and *Pichia* spp. can produce ethanol and form films on the surface, also causing visible spoilage of the product.

To avoid spoilage of raw materials at pre- and post-harvest stages, it is crucial to ensure safe produce handling (for example, by training of the field workers, proper cleaning and disinfection of the harvesting tools and machinery, prevention of injuries during the packaging stage, etc.) as well as appropriate temperature and humidity control.

### 2.3. Processing of Raw Materials

Pre- and post-harvest factors are not the only players when it comes to contamination of processed foods, as the processing environment may also be one of the main sources of contamination, even if quality checkpoints are implemented throughout the production line.

According to Hernández et al. [38], a comprehensive review of published data reveals that the selective pressure of the plant production environment on spoilage yeasts makes production facilities and equipment the primary source of contamination for fruit-based processed foods and beverages. Nonetheless, it is also important to consider other possible sources of contamination, such as air, water, and raw materials.

Processing facilities are abundant in ecological niches in which fungi and yeasts can proliferate, form biofilms, and cross-contaminate processed foods [4]. Equipment and machinery, for example, are possible reservoirs for fungal species if they are not properly disinfected and sanitized regularly [2], as are water systems, which can be reservoirs and vectors of yeast contamination. It is important to note that yeast cross-contamination is more commonly associated with water systems, whereas filamentous fungi contamination is more commonly associated with air systems. This can be explained by the reduced likelihood of airborne spread of yeasts, given that only a small number of them are capable of spore production and only under specific environmental stresses [4,38]. Nevertheless, yeasts can also contaminate air systems if they are carried in aerosols [4].

In terms of ecological niches in machinery, certain species of filamentous fungi and yeasts have been associated with the internal surfaces of processing equipment that come into contact with the product. These surfaces act as barriers or interfaces between the product and the environment, and include ports, valves, filler heads and other machine parts that, although challenging to sanitize, must be disassembled for proper cleaning [4].

One example of this issue is *Geotrichum candidum*, which in the food business is referred to as “machinery mold”, as it causes a dust-like build-up in stainless steel, rubber, or plastic equipment. In food-processing environments, *G. candidum* has been collected from fan blades, air vents, and rubber seals [4]. *Geotrichum candidum* can be found in fresh-cut and processed fruits and vegetables, such as canned tomato paste, blanched and frozen green beans, citrus fruits, and juices, and chopped carrots, which are among the products most susceptible to soft-rot spoilage by this species [56]. If allowed to grown in the food products, *G. candidum* can lead to undesirable flavor changes [4].

Similarly, persistence of yeasts such as *Rhodotorula mucilaginosa*, *Z. rouxii*, *Candida tropicalis*, *Candida glabrata*, and *Kluyveromyces marxianus* in fruit-processing plants has also been reported, particularly in those producing apple concentrates and juices [57].

Water used in processing plants has also been linked to food contamination by spoilage yeasts. In wine factories, for example, *Brettanomyces bruxellensis* and *Candida ishiwadae* have been isolated from washing water, while in fruit processing facilities, washing water has been reported to be contaminated with osmotolerant yeasts such as *Pichia* spp. and *Zygosaccharomyces* spp. [38]. From these examples, it is clear that the microbial quality and organic load of the water used must be carefully managed, and water reuse must follow appropriate rules to ensure that cross-contamination does not occur. Furthermore, contamination from splashes and overspray during cleaning procedures is also a key event in the spread of spoilage yeasts, for example, from floor drains (which are commonly contaminated with high yeast counts) to processing equipment [4]. In this context, measures that can be implemented to reduce the spread of spoilage organisms include the use of an appropriate spray pressure as well as a final disinfectant rinse of processing equipment following floor cleaning.

The air is also a relevant disseminator of fungal spores [32], with ventilation systems being a common reservoir and vector of fungal contamination within factory premises. In this regard, certain manufacturing processes are more susceptible to airborne fungi (such as the filling step), with opportunistic fungi, mainly *Cladosporium*, *Penicillium*, and *Aspergillus*, being the most prevalent in the air and the most challenging threats to eliminate [4].

To prevent fungal air propagation, vacuums have been employed as mold inhibitors in hot filling shelf-stable products such as fruit fillings. However, certain spoilage fungi can overcome this hurdle if residual oxygen is present [2]. In such instances, residual oxygen may facilitate the proliferation of yeasts and filamentous fungi.

Food additives, especially preservatives, should not be used to cover up poor manufacturing practices and lack of sanitization procedures. Nevertheless, industries have tried to manage spoilage microorganisms by adding antimicrobial compounds and preservatives during the manufacturing process, particularly after a fungal deterioration incident affects their product. However, as healthier and safer food requirements have mandated more natural ingredients in processed foods, instead of the usual chemical additives, the food industry has shifted towards using preservative-free clean label products or biopreservation strategies in their foods. On the downside, this can sometimes reduce the stability of the product during storage and therefore increase the likelihood of a food spoilage episode caused by fungi. With this in mind, the critical factor to be ensured in order to prolong product shelf-life and prevent fungal spoilage is environmental hygiene, which includes cleanliness of the entire processing facility, including air and water systems, machinery and work surfaces [32], and the prevention of cross-contamination between raw materials and the finished product [7]. In fact, employee practices and hygiene protocols are the ultimate way to overcome fungal issues and will have a fundamental role in reducing the probability of contamination throughout the production line.

### 2.4. Storage of Final Products

A small number of fungal spores or yeasts introduced into the product (originating from raw materials that have not been eliminated or introduced during the manufacturing process) may cause high levels of contamination and thus irreversible damage and loss of the final stored products [58]. To avoid storage conditions that may allow this problem to occur and to ensure the longest possible shelf-life, appropriate water content and pH levels must be carefully selected for the final product, and the storage temperature (i.e., refrigeration below 8 °C) must be closely monitored. In this regard, researchers have reported water activity and water content as deterministic factors for fungal growth, as temperature, for example, has less effect on growth as long as the water content is below a safe threshold [58,59].

Specific packaging conditions such as modified atmosphere and controlled relative humidity can be implemented to further delay potential fungal spoilage in fruit fillings, even though these should not be considered a substitute for correct storage temperature and low water content [7].

Modified atmosphere packaging (MAP) can be used to provide an optimal atmosphere in packaged foods, including fruit fillings, to extend shelf-life and maintain food quality [60]. This technique encompasses both vacuum packaging (which reduces oxygen in the package headspace to less than 1%) and gas packaging (injection of a specific gas or controlled mixture of gases to replace atmospheric air). In bakery products, carbon dioxide (CO_2_) is commonly used with the purpose of preventing fungal growth by inhibiting the metabolism and interfering with the enzymatic activity of fungi [3,7].

Antimicrobial sachets can also be used as oxygen scavengers or vapor generators (e.g., chlorine dioxide and ethanol), acting as an interactive packaging technology to modify the atmosphere of the microenvironment within a packaged product [7,61].

When selecting the appropriate sachet or the optimum blend of gases, in the case of gas packaging, the physicochemical, microbiological, and sensorial characteristics of the food product must be taken into consideration to ensure the effectiveness of these active food packaging technologies without compromising the sensory profile of the product [7]. For example, already in 1991, Ooraikul et al. [62] highlighted the disadvantage of using ethanol vapor generators for bakery products, as its absorption from the package headspace into the product resulted in sensory rejection of the product, even though this technology was able to control more than ten species of molds, including *Aspergillus* and *Penicillium* species.

The use of MAP goes hand in hand with the control of relative humidity. Relative humidity (RH) is defined as the moisture content (water vapor) of the atmosphere surrounding a food, expressed as a percentage moisture-holding capacity of the atmosphere at a certain temperature and pressure without condensation [63]. Its adequate control within packaging and in finished product storage areas is fundamental, as high humidity levels may foster bacterial growth and food spoilage, whereas low humidity levels can cause products to dry out and lose flavor. Moreover, RH can affect the water activity of foods, as there is an exchange of moisture between the food and its atmosphere until both are in equilibrium.

In storage areas, dehumidifiers of appropriate size (in respect to the rooms’ dimension and moisture load) can be used to control air humidity [64]. For foods, moisture absorbers can be used within packages made of conventional or novel materials [65], and choice of the appropriate packaging (material, presence of micro-perforations, use of scavenging systems, for example), with suitable gas and water vapor barrier properties will minimize moisture transfer between the product and the environment [66,67].

## 3. Prevention Strategies

Although the most common strategies for preventing mold spoilage in fruit fillings for bakery products are pasteurization and the incorporation of chemical preservatives and organic acid salts, other measures can be used to extend the shelf-life of these products. In fact, despite the effectiveness of traditional thermal processing in preserving fruit products, it can negatively affect their nutritional characteristics (vitamins and carbohydrates may be degraded) and sensory appearance (color and flavor) [68]. Moreover, studies have reported that the inhibitory efficacy of various chemical preservatives (such as acetic, propionic and sorbic acids and their salts) is reduced in neutral pH products [69].

**Figure 2:**
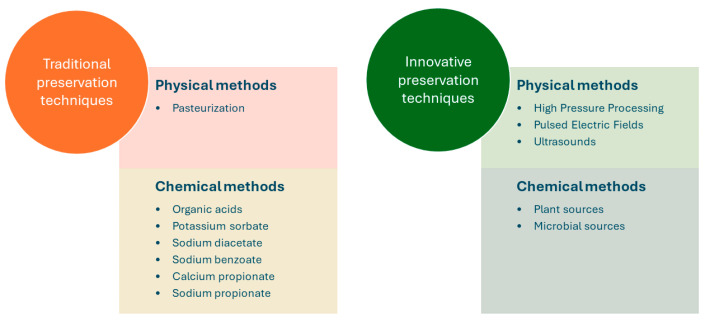
Examples of traditional and innovative preservation techniques for the control of mold spoilage in fruit fillings for bakery products.

In response to the contemporary consumer preference for minimally processed and preservative-free foods, there has been a surge in the development of innovative preservation technologies. These advancements can maintain the freshness of fruit and fruit derivatives while enabling a clean label for the products [1]. These can be categorized into physical methods, which are predominantly non-thermal, and chemical approaches. The following sections review the traditional and novel preservation techniques (Figure 2) that can be applied to fruit fillings.

### 3.1. Traditional Techniques

#### 3.1.1. Physical Method—Pasteurization

Fruit fillings are generally pasteurized to achieve a 5-log reduction in the most resistant microorganisms of public health significance [70]. The treatment can be performed using different time–temperature combinations, depending on the specificities of the product, to inactivate enzymes and vegetative cells that may alter the product or cause disease. However, pasteurization of fruit pulps offers only short-term stabilization, so other preservation methods such as refrigeration must be combined to extend the shelf-life of the product and ensure its quality over time.

**Figure 3:**
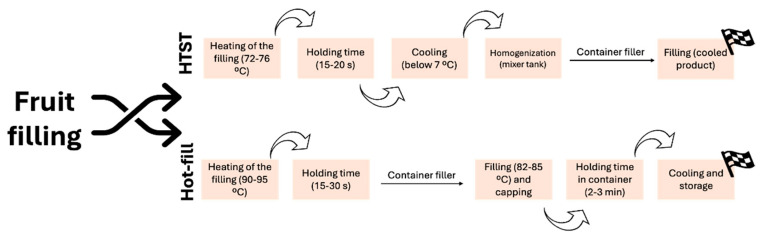
Graphical representation of the differences between HTST pasteurization and hot-filling pasteurization.

In the fruit filling industry, pasteurization is conducted in tanks at the same time as the fruit is mixed and homogenized with other ingredients, and the appropriate time-temperature combination must be selected to ensure the desired characteristics of the product. For example, to ensure the presence of health-promoting polyphenols naturally present in fruit, low temperatures must be used, as these compounds are heat-sensitive and may be lost as a result of the heat treatment [71,72]. For fruit fillings, both HTST (high-temperature short-time) pasteurization and hot-filling techniques are commnly used (Figure 3). Concerning the hot-filling approach, the fruit filling is heated to about 90–95 °C with a holding time of about 15 to 30 s to achieve sterility, and the filling is then introduced to the food product or container [73,74,75]. During the cooling stage, the vacuum will be formed to provide anaerobic conditions to the product [73,74]. Hot-filling has been industrially validated by both microbiological and enzymological parameters [73].

#### 3.1.2. Chemical Preservatives

Chemical preservatives are one of the oldest and most conventional methods of preserving food. The most common antimicrobial agents are organic acids, potassium sorbate, sodium diacetate, sodium benzoate, calcium propionate and sodium propionate (Table 3). 

However, due to health concerns, the use of synthetical chemicals as food additives (e.g., preservatives) has become an increasingly sensitive issue [75]. While organic acids can be used to impair fungal growth, certain fungi, such as *Z. bailii*, exhibit high resistance to these preservatives, particularly weak acids like acetic acid, benzoic acid and sorbic acid. This resistance stems from their ability to use such compounds as carbon sources [83,84,85]. For this reason, besides organic acids, other chemical preservatives have also been extensively used to prevent fungal spoilage and prolong the shelf-life of fruit fillings, including calcium and sodium propionate, potassium sorbate, sodium diacetate and sodium benzoate, among others [7].

Potassium sorbate and sodium benzoate are among the most commonly used chemical preservatives in bakery products with fillings [7]. Potassium sorbate is widely used due to its low cost, ability to prevent spoilage and extend product shelf-life and increased antifungal activity at lower pH values (below 6.0), which is valuable in preserving fruit fillings [7,77]. This preservative has been shown to be more effective against yeasts and molds than bacteria [7]. Similarly, sodium benzoate is also used in the food industry to inhibit the growth of bacteria, yeasts, and molds in acidic food products such as fruit pulp, fruit purées, jellies, and jams, being most effective in products and ingredients with a pH between 2.5 and 4.0 [7,79,86].

While the traditional approach is to add these chemical preservatives as an additional ingredient in the product formulation, recent advances allow them to be added using encapsulation technology, aerosol spraying onto the product, or incorporation into the packaging material [7].

### 3.2. Innovative Techniques

#### 3.2.1. Physical Methods

**Figure 4:**
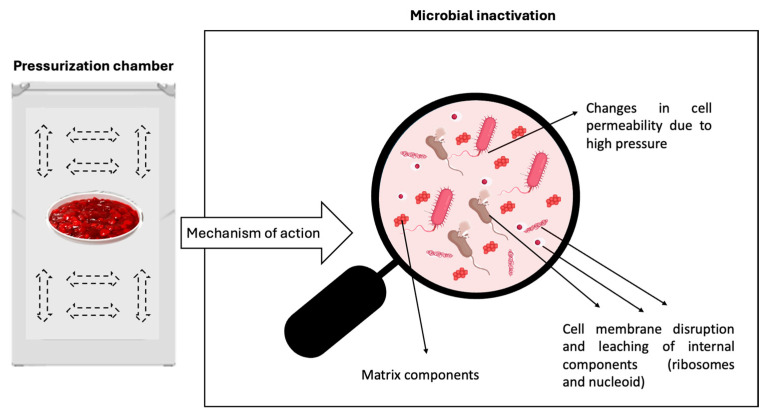
Graphical representation of the microbial inactivation process that occurs during HPP.

**High-pressure processing (HPP).** Generally, HPP uses water as the pressure-transmitting medium in which food products are immersed and subjected to high pressure to reduce their microbial load. Therefore, HPP is also known as high hydrostatic pressure. HPP induces structural damage to microorganisms (cell membrane deformation, leakage from cells, impairment of protein and DNA structures, etc.) while preserving the structure of foods at the macromolecular level (see Figure 4). In this sense, HPP avoids compromising the organoleptic (e.g., color, flavor) and nutritional characteristics of the products [87,88], as is the case when applying traditional methods such as pasteurization. Nevertheless, the effects of HPP on quality characteristics are influenced by pressure level, holding time and temperature, processing patterns, and sample matrices (pH, composition, texture, etc.) [88], so the appropriate parameters to be used in each food matrix must be assessed.

Despite promoting more than 5-log reduction in a variety of vegetative pathogens, one of the main disadvantages of HPP is related to its incapacity to inactivate spores of harmful pathogens, such as *Clostridium botulinum*, at least at room temperature [87]. To solve this issue and inactivate pressure-resistant pathogens and spores, higher temperatures (90–120 °C) must be combined with HPP [89]. Another disadvantage is the specific packaging material required, which must be plastic to ensure a compressibility of at least 15% [87] and is therefore not aligned with the current trend of replacing plastics with more environmentally friendly materials such as biopolymers.

Numerous studies on HPP-treated fruit purées (made of plum, pear, apple, strawberry, nectarine, and banana, for example) focus mainly on the impact of this technology on the quality, nutritional compounds, and enzyme inactivation kinetics of the processed purées [90,91,92]. However, some researchers have also investigated the impact of HPP on the microbiological quality of the products. Marszałek et al. [93] observed that HPP at 300 MPa or 500 MPa (at either 0 or 50 °C, for 15 min) reduced yeast and mold counts from 4.6 log CFU/g and 3.8 log CFU/g, respectively, to less than 1 log CFU/g. Another study by Evelyn and Silva [94] studied the inactivation of *Bys. nivea* ascospores in strawberry purée using high pressure (600 MPa) at 75, 60, 50 and 38 °C for 10 min and observed reductions of 1.4 log, 0.8 log, 0.7 log, and 0.5 log CFU/mL, respectively.

**Pulsed electric fields (PEFs).** PEF processing involves placing the food between two electrodes, typically at room temperature, and applying brief (micro- to milliseconds) bursts of high-voltage electricity (20–80 kV) that create electric fields, leading to electroporation of microbial cell membranes and, consequently, microbial inactivation [95,96].

Several studies have investigated fungal and microbial inactivation by PEFs on fruit juices, smoothies, eggs, and milk and milk-derived products, with successful results [97,98,99,100,101]. To our knowledge, on the other hand, studies on the effect of PEFs on fruit fillings and purées are limited, in particular those focusing on yeast and mold inactivation, thus suggesting a research gap in the literature. Geveke et al. [102], for example, tested the effect of PEF processing on *E. coli* populations in strawberry purée and observed a reduction of 7.3 log CFU/mL when applying an electric field strength of 24 kV/cm at 52.5 °C, but its effect on fungal contamination was not tested.

The main advantages of PEF processing include the maintenance of the original sensory and nutritional characteristics of foods (due to the brief processing time and low temperatures), reduced energy consumption (compared to conventional thermal methods), and non-production of waste [103]. On the other hand, limitations of PEF are linked to the unavoidable occurrence of electrochemical reactions and chemical processes at the electrode–food boundary of a PEF treatment chamber [104]. These reactions may lead to corrosion and fouling of the electrodes, water electrolysis, migration of electrodes’ material components, and chemical deviations in the food product, thus calling into question the safety, quality, process efficiency, equipment reliability, and cost-effectiveness of this technology [104].

**Ultrasound.** Functioning within the frequency range of 20 to 100 kHz and employing a higher sound intensity between 10 and 1000 W/cm^2^ [105], this method involves the transmission of ultrasonic waves in a liquid. These waves induce the rapid formation and collapse of small bubbles (cavitation) with high localized temperatures and pressure, leading to the breakdown of cell walls, disruption of cell membranes, and damage to DNA [106,107]. As well as inactivating vegetative cells, this technology is capable of eliminating microbial spores and inactivating enzymes [108,109,110].

While research on the effects of sonication on fungal contamination in fruit purées and fillings is scarce, several works have reported encouraging results regarding this technique applied to molds and yeast inactivation for fruit juice preservation [108]. Nevertheless, the use of ultrasounds at high intensities may present some drawbacks. In the context of fruit fillings, a considerable disadvantage would be the generation of heat, due to an escalation in temperature, which would negatively affect the organoleptic and nutritional properties of the product [102].

#### 3.2.2. Chemical Methods

**Plant sources.** Plants may present antifungal activity as a result of the bioactive compounds (secondary metabolites) they contain, such as alkaloids, phenols, flavonoids and terpenoids, which alone or synergistically inhibit the growth of phytopathogenic fungi [30,111].

Plant extracts and plant essential oils, which are rich in such bioactive compounds, can be produced using a variety of solvents and extraction methods [30], and the extracts, essential oils, or purified compounds can be applied to numerous food matrices. For example, Teixeira et al. [112] observed the effectiveness of garlic peel extract in controlling the proliferation of *Colletotrichum acutatum* in apples. Similarly, Elshafie et al. [113] successfully applied thyme and vervain essential oils against three postharvest fungal pathogens of peach fruits, namely *Monilinia laxa*, *Monilinia fructigena*, and *Monilinia fructicola*, with significant reduction of the brown rot lesion diameter. In turn, Ochoa-Velasco et al. [114] coated mango and papaya with edible films containing a mixture of thymol and carvacrol and evaluated their effectiveness against *Colletotrichum gloeosporioides*, reporting a fungistatic effect due to a reduction of lesions in coated fruits. More specific to our research question, Ribes et al. [115] reported on the antifungal capacity of clove and cinnamon oil/water emulsions to reduce or inhibit the growth of *A. flavus*, *A. niger* and *P. expansum* in strawberry jams.

The antifungal activity promoted by plant extracts, essential oils, and their derivatives can be attributed to a variety of mechanisms, including mutagenic activity (reduction of gene expression), enzyme inhibition, cytotoxic effects on eukaryotic cells, permeabilisation of the cell wall and cytoplasmic membrane, disruption of the proton motive force, electron flow and active transport, and clotting of cell contents [30].

Counteracting with the advantages of their use, plant-derived preservatives face some challenges, such as the weak stability and sensitivity of bioactive compounds to processing and storage conditions (which may lead to loss of bioactivity) [116]; strong odor (particularly in the case of essential oils) even when low concentrations are used, which may hinder their use in food matrices [117]; and possible interactions with food components (i.e., carbohydrates, lipids, proteins) that may occur and result in reduced biological activities (compared to results of in vitro studies) [118]. In the latter case, the literature on the antifungal activity of plant extracts, essential oils, and derivatives in carbohydrate-rich matrices such as fruit fillings is scarce and inconsistent. For example, Gutierrez et al. [118] reported a reduction in the efficacy of oregano and thyme essential oils against *L. monocytogenes* at 5% and 10% starch concentrations, in contrast to the observation by Shelef et al. [119] that carbohydrates in foods do not have a protective effect on bacteria against the action of essential oils.

**Figure 5:**
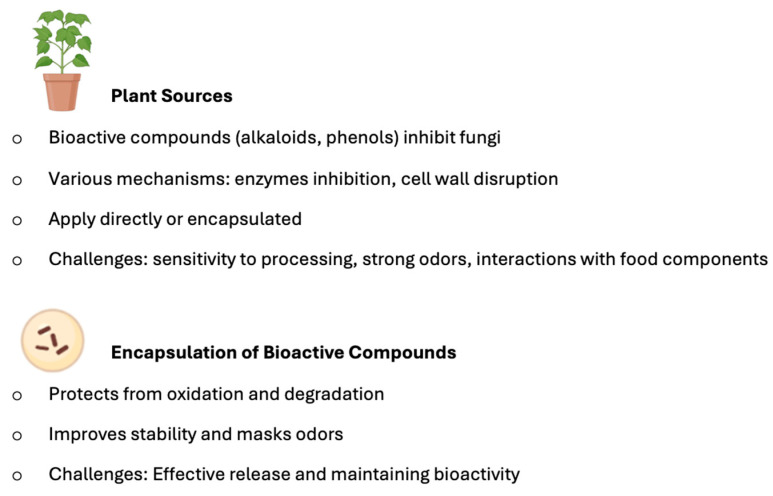
Overview of plant sources and encapsulation of bioactive compounds as strategies for preservation of fruit fillings.

Encapsulation of bioactive compounds and essential oils could be a solution to the above-mentioned issues. Micro- and nano-encapsulation using suitable vehicles are adequate strategies for improving the stability of compounds, avoiding their oxidation and degradation, reducing interactions with food components, and masking off-flavors and odors [117]. Figure 5 presents a brief summary of the information described in this section.

**Microbial sources**. While plants offer a broad range of promising antimicrobial options, these are often seasonal, and their composition varies depending on the climate, cultivation method, and plant development stage, among other factors. On the other hand, microorganisms are a natural source that can be easily isolated in controlled environments with higher production rates [120].

In recent years, peptides obtained from microbial sources have attracted attention as natural preservatives with antimicrobial and antifungal potential, considering their inhibitory effect on common pathogenic bacteria and fungi, yeasts, filamentous fungi (e.g., *A. flavus*) and mold in foods [121] and their stability in both acidic and alkaline environments and at high temperatures [1]. These characteristics suggest that antimicrobial peptides have the potential to replace chemical preservatives in a wide range of products [1].

**Figure 6:**
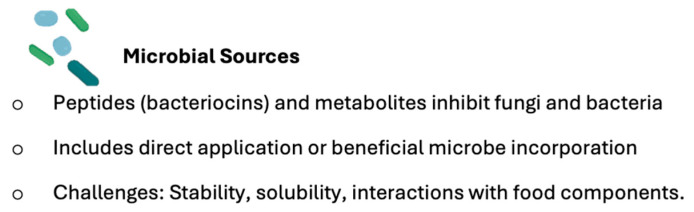
Overview of microbial sources as a strategy for the preservation of fruit fillings.

Bacteriocins, for example, are among those peptides with diverse biological activities, including antimicrobial and antifungal activity [1] (Figure 6).

To date, numerous studies have successfully isolated and identified bacteriocins synthesized by gram-positive and gram-negative bacteria, with *Lactobacillus*, *Pediococcus*, and *Leuconostoc* species reported to be the most commonly associated with antifungal bacteriocins [1,122,123]. The findings of several of these studies have been compiled in databases to facilitate the automated identification of bacteriocins and their genetic determinants from genomic data [123,124].

Salivaricin KLD, for instance, is a bacteriocin produced by *Lactobacillus salivarius* KL-D4 (currently named *Ligilactobacillus salivarius*) that has been studied for its use in the biopreservation of bakery cream fillings (cream or custard fillings containing egg or a dairy product) [69]. This peptide is heat-tolerant and stable over a wide pH range and shows effective inhibitory activity against *Pseudomonas* spp. (e.g., *Pseudomonas stutzeri*) and some strains of *Enterococcus faecalis* [69].

Other metabolites also produced by lactic acid bacteria include organic acids (lactic, acetic, propionic and phenyllactic acids), fatty acids, carboxylic acids, reuterin, and cyclic peptides such as nisin, which is produced by some *Lactococcus lactis* strains but has no relevant antifungal activity [122,125].

On the other hand, natamycin, produced by *Streptomyces natalensis*, is an effective antifungal peptide against most mold and yeasts (including *Candida* spp., *Aspergillus* spp., *Cephalosporium* spp., *Fusarium* spp. and *Penicillium* spp.) [120]. It is widely used as a preservative in cheeses, yogurts, sausages, juices, wines, etc., with the particular advantages of being listed by the FDA as GRAS (generally recognized as safe) and being odor- and colorless [120]. The antifungal action of natamycin is related to its high affinity for ergosterol, a component of the eukaryotic cell membrane which provides binding sites for natamycin; the two compounds form a polyene–sterol complex capable of modifying membrane permeability, resulting in rapid leakage of solutes and consequent interruption of growth [1,120,126].

Nevertheless, the effectiveness of any antimicrobial peptide must be carefully investigated, as there may be adsorption to food components, poor solubility, enzymatic degradation, irregular distribution in the food matrix, and interactions between these natural additives and food components, food characteristics (pH, texture, etc.), and processing techniques; therefore, the appropriate application conditions must be optimized [123].

Another biocontrol strategy involves incorporating selected microbes into the food product instead of the purified metabolites. In this way, the selected bacterial and/or fungal species can antagonize the spoilage microorganisms via competition for nutrients, biofilm formation, and production of volatile bioactive metabolites, toxic proteins, and antifungal enzymes, for example [30,123].

For application in bakery products, lactic acid bacteria are among the main microbes tested against fungi (e.g., *Lactiplantibacillus plantarum*, *Levilactobacillus brevis*) [1]. For fruit bioprotection, bacteria, especially from the *Bacillus* group (e.g., *B. amyloliquefaciens*, *B. megaterium*, *B. subtilis*), and some yeasts (e.g., *R. mucilaginosa*, *Saccharomyces cerevisiae*, *Candida oleophila*, *Debaryomyces hansenii*) have been used [1,30]. In a study by Arrarte et al. [127], for example, the growth of *P. expansum* in apples was inhibited by the yeast *D. hansenii*, which exhibits psychrotrophic properties and produces hydrolytic enzymes. Another example is provided in the study by Wei et al. [128], in which strawberries were sprayed with *Cryptococcus laurentii* 6, 3, and 0 days before harvest, resulting in a 22% reduction in the incidence of *Botrytis cinerea* compared to controls.

It appears that a myriad of antifungal microbes and/or metabolites are available to delay fungal growth on fresh fruits during transport and storage, preventing the spoilage issue earlier in the production process and reducing potentially higher contamination levels in the final product [122]. However, it is important to note that to date, few antagonistic yeasts are commercially available for use as biocontrol agents. For this reason, further studies are needed to better understand the interactions between antagonistic yeasts, contaminant molds, and fruit and environmental characteristics in order to develop successful formulations that are also economically viable [129].

## 4. Conclusions

Fruit fillings are a perfect environment for microbial growth and fungal spoilage, contributing to food waste, food recall, and economic losses when antiproliferative strategies are not put into place. In order to prevent fungal spoilage throughout the production process, this work has clarified the matrix characteristics and physicochemical properties of fruit fillings intended for pastry products as well as the main issues that can promote fungal growth during each of the manufacturing steps, most notably during the handling of raw materials in the pre- and post-harvest periods, during product processing, and during storage of the finished product.

Considering the upsurge of the “clean label” trend and in order to avoid traditional preservation strategies such as pasteurization and the addition of synthetic chemical additives, this work also discussed novel techniques considered suitable for inhibiting fungi proliferation in fruit fillings: high-pressure processing, pulsed electric fields, ultrasounds, the use of plants and their extracts, essential oils and bioactive compounds, and microorganisms and their metabolites.

In addition to good manufacturing practices and hygiene protocols, which are non-negotiable, the combination of one or more innovative preservation techniques, low storage temperatures, reduced water activity, and pH values creates a hurdle effect capable of reducing the risk of fungal spoilage in the final product. This is crucial given that many fruit-based products used as ingredients in pastry and bakery items do not undergo further processing and are considered ready-to-eat products.

Nevertheless, further research is still needed to ensure the successful application of these novel preservation strategies. In particular, optimization for each food product is essential, as each individual matrix’s characteristics and potential interactions with novel additives must be considered.

## Figures and Tables

**Table 1:** Food spoilage issues and the associated mold, contamination source, and mycotoxin production potential in different products.

Product Type	Spoilage Microorganisms	Contamination Source	Spoilage	Mycotoxin Production Potential	References
Shelf-stable, hot-filled products	*Aspergillus*	Raw material storage and air of the food processing facility	Visible deposits of black conidia on surface	Yes	[4,31,32,33]
	*Penicillium*		Small patches of growth that may be hard to visualize	Yes	[4,33,34]
	*Cladosporium*		Product discoloration	Yes	[11,33]
Stored fresh fruit and processed foods with low-to-medium water activity	*Mucor*	Raw material	Aerial hyphae of mucoralean fungi visible on product surface; product fermentation with gas production	Yes	[4,33,35]
	*Rhizopus*			Yes	
Stored fresh fruit, processed fruit products	*Botrytis*	Raw material; air of the food processing facility	Gel formation	No	[18,36]

**Table 2:** Food spoilage issues and the associated yeast and contamination source in different fruit/high-sugar products.

Product Type	Spoilage Microorganisms	Contamination Source	Spoilage	References
Processed fruit products	*Pichia*	Raw material	Off-flavor and gas production due to fermentation	[16,37]
High-sugar-content products	*Zygosaccharomyces*	Raw material	Visible growth on the surface, fermentation, off-flavor, and off-odors	[38,39,40]
Processed fruit products	*Saccharomyces*	Raw material	Production of ethanol and film formation on surface	[37,38,39]
Processed fruit products	*Candida*	Raw material, lack of effective hygiene protocols	Film formation on surface	[4,37,39]
Processed fruit products and products stored at low temperatures	*Rhodotorula*	Airflow, vectors, processing facility equipment	Visible red colonies on product surface without fermentation	[37,38,39,40]

**Table 3:** Most common chemical preservatives used in fruit fillings and their mechanism of action and uses.

Preservative	Mechanism of Action	Uses	Reference
Organic acids	Decreasing bacterial intracellular pH value	Used as emulsifiers, stabilizers, preservatives, flavour enhancers, and firming agents	[76]
Potassium sorbate	Modifications to the structure and functionality of the bacterial cell membrane, as well as suppression of metabolic activity and transport functions	Bacteriostatic and fungistatic agents in a variety of processed food	[77]
Sodium diacetate	Decreasing bacterial intracellular pH value	Used as a flavouring agent/adjuvant for control of pH and as an antimicrobial preservative	[78]
Sodium benzoate	Decreasing bacterial intracellular pH value and inhibition of anaerobic fermentation of glucose	Antimicrobial preservative	[79]
Calcium propionate	Inhibition of enzymes necessary for metabolism and decreasing bacterial intracellular pH value	Antimicrobial preservative	[80,81]
Sodium propionate	Inhibition of enzymes necessary for metabolism and decreasing bacterial intracellular pH value	Antimicrobial preservative	[81,82]

## Data Availability

No new data were created or analyzed in this study. Data sharing is not applicable to this article.
